# *Magnaporthe oryzae* Transcription Factor MoBZIP3 Regulates Appressorium Turgor Pressure Formation during Pathogenesis

**DOI:** 10.3390/ijms23020881

**Published:** 2022-01-14

**Authors:** Chengyu Liu, Ningning Shen, Qian Zhang, Minghui Qin, Tingyan Cao, Shuai Zhu, Dingzhong Tang, Libo Han

**Affiliations:** College of Life Science, State Key Laboratory of Ecological Control of Fujian-Taiwan Crop Pests, Key Laboratory of Ministry of Education for Genetics, Breeding and Multiple Utilization of Crops, Plant Immunity Center, Fujian Agriculture and Forestry University, Fuzhou 350002, China; liuchengyufafu@163.com (C.L.); shenningning1994@163.com (N.S.); 15739335705@163.com (Q.Z.); qinminghui657@163.com (M.Q.); cty18855792177@163.com (T.C.); shuaiz22620@126.com (S.Z.); dztang@fafu.edu.cn (D.T.)

**Keywords:** *Magnaporthe oryzae*, transcription factor, appressorium turgor pressure, virulence

## Abstract

The devastating fungus *Magnaporthe oryzae* (*M. oryzae*) forms a specialized infection structure known as appressorium, which generates enormous turgor, to penetrate the plant cells. However, how *M. oryzae* regulates the appressorium turgor formation, is not well understood. In this study, we identified *MoBZIP3*, a bZIP transcription factor that functioned in pathogenesis in *M. oryzae*. We found that the pathogenicity of the *MoBZIP3* knockout strain (Δ*mobzip3*) was significantly reduced, and the defect was restored after re-expression of *MoBZIP3*, indicating that *MoBZIP3* is required for *M. oryzae* virulence. Further analysis showed that *MoBZIP3* functions in utilization of glycogen and lipid droplets for generation of glycerol in appressorium. *MoBZIP3* localized in the nucleus and could bind directly to the promoters of the glycerol synthesis-related genes, *MoPTH2*, *MoTGL1* and *MoPEX6*, and regulate their expression which is critical for glycerol synthesis in the appressorium turgor pressure generation. Furthermore, the critical turgor sensor gene *MoSln1* was also down regulated and its subcellular localization was aberrant in Δ*mobzip3*, which leads to a disordered actin assembly in the Δ*mobzip3* appressorium. Taken together, these results revealed new regulatory functions of the bZIP transcription factor MoBZIP3, in regulating *M. oryzae* appressorium turgor formation and infection.

## 1. Introduction

Rice blast disease caused by the devastating fungus *Magnaporthe oryzae* (*M. oryzae*) is the greatest threat to the global rice harvest and leads to serious economic losses worldwide every year [1]. Rice blast are spread by wind or splash dispersal of the conidias to the host surface [2]. After the conidias landing and adhering to the rice leaf cuticle, *M. oryzae* will undergo extensive developmental changes while building elaborate infection structures, invading plant cells, and finally proliferating inside the host cells [3,4]. The conidias quickly germinate and form a dome-shaped infection structure at the tip called appressorium with great internal turgor, which is required for cuticle penetration [5]. Thus, developing a mature appressorium is an essential prerequisite for *M. oryzae* to successfully invade the plant cells.

The appressorium initiates and develops at the germ tube tips. It accumulates multiple and high concentrations of metabolites which generates enormous turgor pressure (>eight MPa) [6,7]. Relying on the turgor pressure, a narrow penetration hypha develops from the base of the appressorium and mechanically punches the plant cuticle [8]. Since the differentiation of infection structures is without exogenous nutrients, it underlines the fact that appressorium development is nutrified by compounds transferred from the conidia. The most abundant solute in appressorium is glycerol and the other polyols, and the germinating conidia utilize intrinsic glycogen and lipid droplets, to provide sufficient energy and materials for appressorial morphogenesis and turgor generation [5,7]. The turgor-sensing histidine–aspartate kinase, Sln1, could enable the appressorium to sense the increasing turgor and then intrigue a set of downstream signals in the appressorium for facilitating penetration of the host cells [9]. Thus, complex enzymatic catalysis, cellular events and signaling are involved in appressorium turgor formation and regulation. Currently, we have not totally understood the regulation mechanism in these processes.

Transcription factors generally include the BZIP proteins, MYB-like proteins, MADS-box proteins, helix-loop-helix proteins, zinc-finger proteins, and homeobox proteins [10]. They could bind to specific promoter sequences to regulate expression of the target genes and function as key regulatory elements in the control of gene expression in development and plant infection in fungi [11]. The BZIP proteins belong to one of the largest transcription factor families and are widely distributed in eukaryotes. Members of this family play a diverse variety of regulatory roles in fungi. For example, the BZIP protein FlbB of *Aspergillus nidulans* (*A. nidulans*) and *Aspergillus fumigatus* (*A. fumigatus*) functions in asexual development [12,13]. And MetR protein from *A. nidulans* was reported to be critical in fungal development through regulation of sulfur metabolism [14]. In *M. oryzae*, over twenty members of BZIP proteins are encoded in the genome, and the members such as MoAP1, MoBZIP13, MoBZIP22, MoMetR, MoBZIP5 and MoMeaB were shown to play critical roles in *M. oryzae* sporation, appressiorium formation or host infection [15,16,17]. These findings provided new insights for understanding the crucial roles of bZIP proteins in both development and pathogenesis in *M. oryzae*.

In this study, we transcriptionally analyzed the *M. oryzae* *BZIP* genes in strain Y34 which was isolated in a field of the Yunnan province of China [18,19]. A total of 22 *BZIP* genes were found through BLAST search and of which, the transcripts of *MoBZIP3*, *MoBZIP16*, *MoBZIP17*, *MoBZIP20*, *MoBZIP23*, *MoBZIP24* were increased in abundance in Y34 at appressorium or host infection stages. Deletion of each of these six *BZIP* genes showed that only Δ*mobzip3* exhibited an obviously weaker virulence than the control, indicating that this protein plays a role in Y34 pathogenesis. Infection analysis showed that Δ*mobzip3* was compromised in rice sheath cell penetration. Furthermore, utilization of glycogen and lipid droplets in appressorium was disordered and glycerol accumulation in Δ*mobzip3* appressorium was decreased, suggesting that the turgor pressure was compromised. MoBZIP3 could directly bind the promoters of the glycerol synthesis-related genes, such as *MoPTH2*, *MoTGL1* and *MoPEX6* and regulated their expression. In addition, *MoBZIP3* also functions in the appressorium turgor sensor protein, *MoSln1*-mediated turgor recognition. The expression of *MoSln1* was reduced and the protein subcellular localization was changed in Δ*mobzip3*. These resulted in an abnormal assembly of actin ring in the specialized cell which is critical for the host penetration of *M. oryzae*. In conclusion, we demonstrated the biological functions of the bZIP protein in regulation of both glycerol synthesis and *MoSln1* mediated-turgor sensing in appressorium.

## 2. Results

### 2.1. Identification of BZIP Transcription Factor Genes in Y34

**Figure 1:**
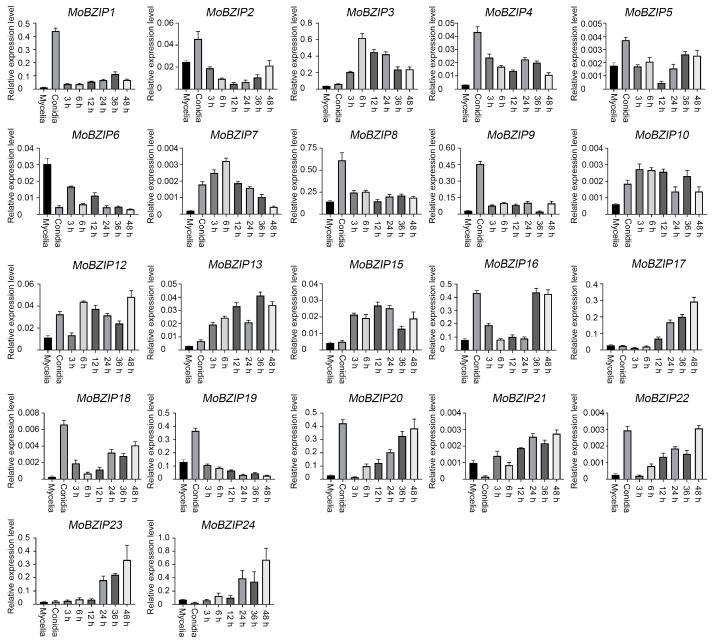
Expression profiles of *BZIP* genes in Y34. Y34 conidia (1 × 10^5^ conidia/mL) was sprayed on rice leaves. mRNAs were extracted at different infection stages (3, 6, 12, 24, 36 and 48 h after spray, respectively). mRNAs from conidia and mycelia were also included in this experiment. cDNAs were then generated using these mRNAs. Quantitative real-time PCR analysis of the expression of *BZIP* genes was conducted. The experiments were repeated three times with similar results. Error bars represent ±SE of three biological replicates. Primers used in this experiment were listed in Supplemental Table S1.

Since the genome of *M. oryzae* Y34 was sequenced and annotated [19], We searched and blast the BZIP domain in the NCBI Genome Database (www.ncbi.nlm.nih.gov/genome) (accessed on 10 August 2021). A total of 22 *BZIP* genes were found in Y34 genome. Compared with the annotated *BZIP* genes in isolates of Guy11 and KJ201, no *MoBZIP11* and *MoBZIP14* were present in the Y34 genome, but additional two *BZIP* genes (*MoBZIP23* and *MoBZIP24*) were identified [15,16]. We then analyzed the expression profiles of these *BZIP* genes in Y34 through quantitative real-time PCR analysis. As shown in Figure 1, *MoBZIP1*, *MoBZIP4*, *MoBZIP8*, *MoBZIP9*, *MoBZIP18* (*MoHAC1*) and *MoBZIP19*, showed the highest transcription levels in conidium stage than those of other stages. And in the infection stage, *MoBZIP3*, *MoBZIP12* (*MoMEAB*), *MoBZIP13* (*MoHAPX*), *MoBZIP16*, *MoBZIP17* (*MoATFA*), *MoBZIP20*, *MoBZIP23* and *MoBZIP24* showed relative higher expressions than in the mycelial or conidium stage. These results suggested that *BZIP* genes in Y34 were differently expressed and may play potentially various roles at developmental or infection stages.

### 2.2. MoBZIP3 Plays a Role in M. oryzae Infection

To characterize the function of *BZIP* genes in *M. oryzae*, we next carried out gene replacement experiments to knockout genes which expressed at the infection stages in *M. oryzae*. A total of eight genes were revealed to express at a high level at the infection stages (Figure 1). We successfully knocked out six genes including *MoBZIP3*, *MoBZIP16*, *MoBZIP17*, *MoBZIP20*, *MoBZIP23* and *MoBZIP24* except for *MoBZIP12* and *MoBZIP13,* though we repeated for several times. All deletion mutants were confirmed by polymerase chain reaction (PCR) using sets of PCR primers (Supplemental Figure S1).

**Figure 2:**
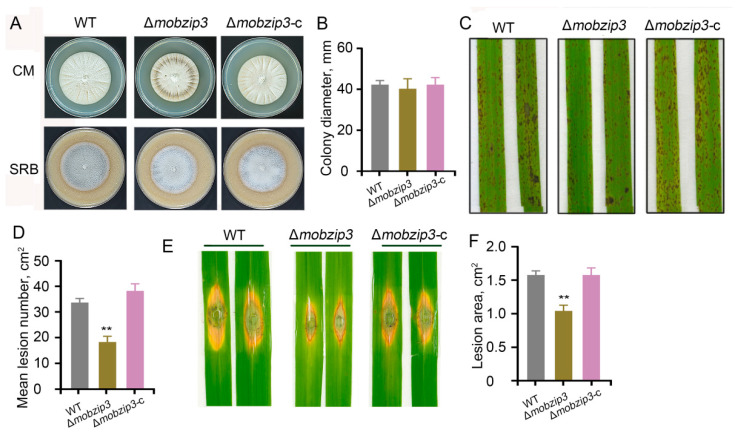
Pathogenesis analysis of mutants of *BZIP* genes. (**A**), The same numbers of conidia of WT, Δ*mobzip3* and the complemented strain (Δ*mobzip3*-c) were cultured on the completed mediate (CM) and straw rice bran (SRB) medium. Images showing seven-day-old cultures of the indicated *M. oryzae* on the two types of mediums. (**B**), Colony diameter of the indicated *M. oryzae* in (**A**). (**C**), Pathogenesis analysis of these indicated *M. oryzae* using conidia spraying (**C**) or punched (**E**) inoculated methods to infect rice leaves (*O. sativa* cv. Nipponbare). Images were obtained from 5 days after inoculation. The experiments were repeated three times with similar results. (**D**,**F**), Quantification of the lesion numbers (**C**) and area (**E**) of the rice leaves shown in (**C**) and (**E**), respectively. Error bars represent SD (*n* = 20) and asterisks (**) represent a significant difference (*p* < 0.01).

Next, we cultured these deletion mutants in the complete medium (CM) and straw rice bran (SRB) medium. We found that none of the knockout mutants exhibited an obvious change in growth rate when compared to the wild type (WT) (Figure 2A,B; Supplemental Figure S2A). We then analyzed the pathogenesis of these mutants using the same amounts of conidias to infect the rice leaves (*Oryza sativa* cv. Nipponbare). Rice seedlings were sprayed with conidias of these *M. oryzae* mutants and only mutant of *MoBZIP3* (Δ*mobzip3*) displayed decreased pathogenicity. It developed an obvious decreased number of lesions at the rice leaves than the other types of *M. oryzae*. And the complemented strain (Δ*mobzip3*-c) could recovered its pathogenecity (Figure 2C,D; Supplemental Figure S2B,D). Punch inoculated assay also showed that infection ability of Δ*mobzip3* was decreased (Figure 2E,F; Supplemental Figure S2C,E). In all, these results indicate that *MoBZIP3* is required for full pathogenicity of *M*. *oryzae* and its deletion reduces virulence.

### 2.3. MoBZIP3 May Involve in Appressorium Turgor Formation

**Figure 3:**
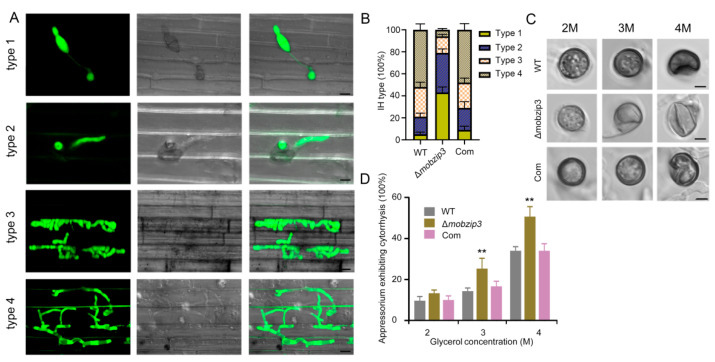
Defects of the Δ*mobzip3* mutant in plant infection. (**A**), GFP labeled *M. oryzae* were used to infect rice sheath cells. Images showing the four types of invasive hyphae (IH) in rice cells (type 1, no hyphal penetration; type 2, IH with fewer than two branches; type 3, IH with more than two branches; and type 4, IH that fully occupies a plant cell and moves into neighboring cells) Bar = 20 μm. (**B**), Growth of the IH in rice cells was quantified and statistically analyzed at 36-h post-inoculation for WT, Δ*mobzip3* and the complemented strain. Error bars represent SD, *n* ≥ 50 cells. (**C**), Appressorium turgor analysis using an incipient cytorrhysis (cell collapse) assay. Representive images showing the conidia of WT, Δ*mobzip3* and the complemented strain were put in glycerol (2–4 M) for 10 min. Bar = 5 μm. (**D**), Quantification of the collapsed cells in (**C**). The percentage of collapsed appressorium was recorded by observing at least 100 appressorium and the experiment was repeated three times. Error bars represent standard deviation and asterisks (**) represent a significant difference (*p* < 0.01).

Penetration assays using rice sheath tissues were carried out to investigate how *MoBZIP3* functions in *M. oryzae* pathogenesis. It has been defined that four types of invasive hyphal (IH) could be classified during host infection: (type one, appressorium formation and no hyphal penetration; type two, IH with less than two branches; type three, IH with more than two branches; type four, IH that fully occupies a plant cell and penetrates into neighboring cells) [20]. To further observe this process, we labeled the WT, the Δ*mobzip3* and the complemented strains with GFP (Figure 3A). Penetration assays were then conducted by observing 100 appressorium for each GFP labeled *M. oryzae* strain and classifying their IH types at 36 h after infection. We found that in WT the four types of IH were 3.3%, 17.7%, 26.5% and 52.5% IH respectively. In contrast, those in the Δ*mobzip3* were 42.6%, 35.4%, 14.5%, and 7.5% (Figure 3B). These results indicated that type 1 IH in Δ*mobzip3* occupies the most part and penetration of Δ*mobzip3* were delayed at the early infection stages.

The penetration assay above indicated that the appressorium penetration ability was decreased in Δ*mobzip3*. This prompted us to evaluate the turgor pressure inΔ*mobzip3* appressorium. The incipient collapse assay [21] showed that the collapse rate of appressorium of Δ*mobzip3* upon glycerol treatment was significantly higher than the WT and the complemented strain (Figure 3C,D), suggesting that turgor pressure of Δ*mobzip3* appressorium was decreased compared with that of the WT strain.

### 2.4. MoBZIP3 Functions in Utilization of Glycogen and Lipid Droplets in Appressorium

**Figure 4:**
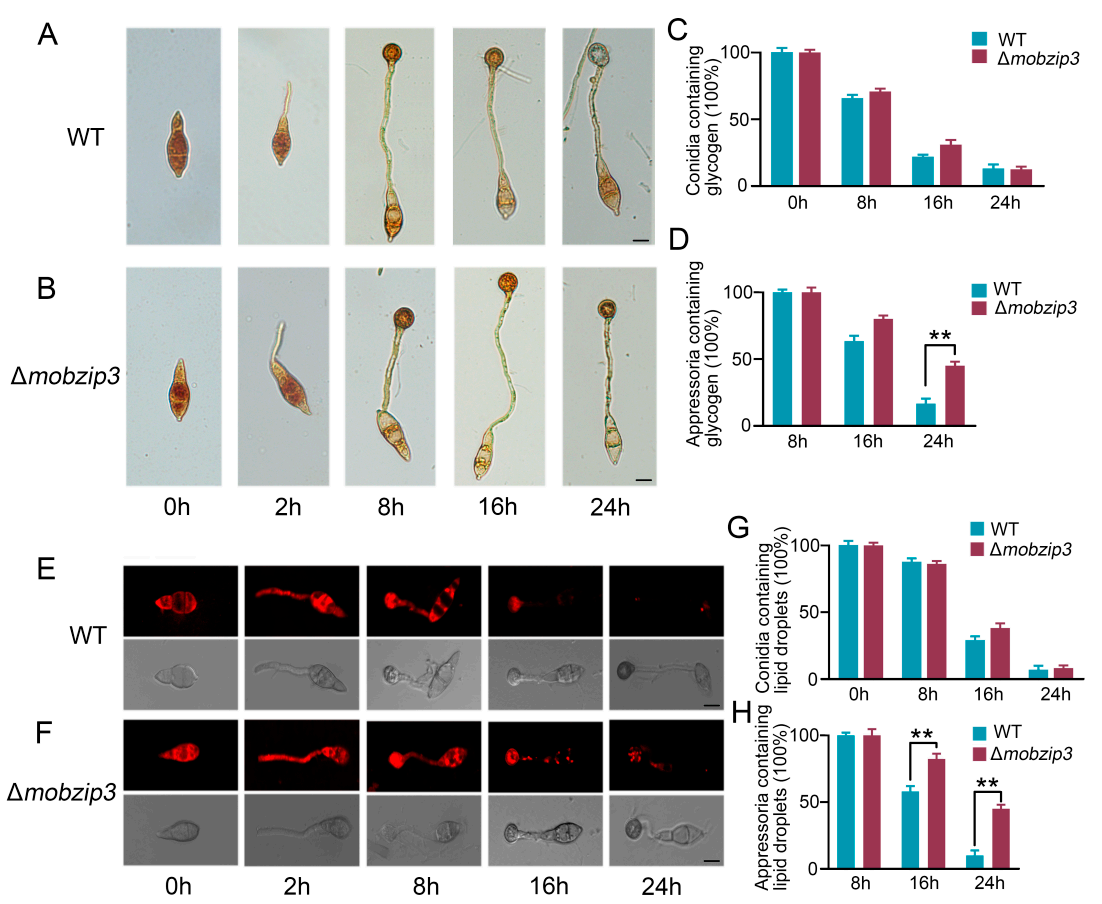
*MoBZIP3* affects utilization of glycogen and lipid droplets in appressorium. Images showing the distribution of glycogen in WT (**A**) and Δ*mobzip3* (**B**) during appressorium development. Bar = 5 μm. Samples were stained with KI/I2 solution. The percentage of glycogen in conidia (**C**) and appressoruin (**D**) during appressorium development. Error bars represent the standard deviation. Significant differences of Δ*mobzip3* compared with the WT strain were estimated by students *t* test, *n* ≥ 50 cells, ** *p* < 0.01. Images showing the distribution of lipid droplets in WT (**E**) and Δ*mobzip3* (**F**) during appressorium development. Samples were stained with Nile red and observed with fluorescence microscopy. The lipid droplets show a red signal fluorescence. Bar = 10 μm. The percentage of lipid droplets in conidia (**G**) and appressoruin (**H**) during appressorium development. Error bars represent the standard deviation. Significant differences of Δ*mobzip3* compared with the WT strain were estimated by Student’s *t*-test, *n* ≥ 50 cells, ** *p* < 0.01.

Glycerol accumulation generated the turgor pressure in the appressorium and production of glycerol was mainly from transfer and utilization of glycogen and lipids in the conidia [22,23]. Thus, to investigate the glycogen distribution and lipid turnover of Δ*mobzip3*, we stained glycogen and lipids using potassium iodide and Nile red in appressorium, respectively. We found that at eight h after conidia developed on a hydrophobic surface, glycogen was transferred from conidia to appressorium in WT. And then the amount of glycogen in appressorium gradually decreased. In contrast, in Δ*mobzip3* appressorium, the amount of glycogen did not decrease significantly as compared with WT. Even at twenty four h appressorium, about 50% glycogen was not transferred (Figure 4A–D). Similarly, the distribution of lipids droplets exhibited the same pattern as glycogen in Δ*mobzip3*, as shown in Figure 4E–H. In all, these results indicated that *MoBZIP3* is required for lipid droplets and glycogen utilization in appressorium development.

### 2.5. MoBZIP3 May Function as a Working Transcription Factor in Regulating Glycerol Synthesis Genes Expression

**Figure 5:**
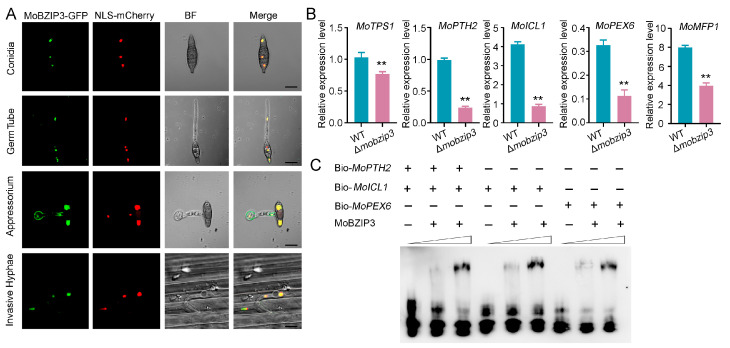
*MoBZIP3* may function as a transcription factor. (**A**), Localization analysis of *MoBZIP3*. *MoBZIP3*-GFP driven by its native promoter was expressed in NLS-mCherry labeled Δ*mobzip3*. Both GFP and mCherry were observed under fluorescence microscopy in the *M. oryzae* conidia, germ tube, appressorium and invasive hyphae. Bar = 10 μm. (**B**), Quantitative real-time PCR analysis expression of *MoTPS1*, *MoPTH2*, *MoICL1*, *MoPEX6*, *MoMFP1* in WT and Δ*mobzip3*. The experiments were repeated three times with similar results. Error bars represent ±SE of three biological replicates. Asterisks represent a significant difference (** *p* < 0.01). Primers used in this experiment were listed in Supplemental Table S1. (**C**), EMSA showing *MoBZIP3* could directly bind to the promoter fragment of *MoPTH2*, *MoICL1* and *MoPEX6*.

We next investigated the localization of *MoBZIP3*. *MoBZIP3*-GFP driven by *MoBZIP3* native promoter was expressed in the nuclear localization signal (NLS)-mCherry labeled Δ*mobzip3* mutant. Localization of *MoBZIP3* was then observed in the *M. oryzae* conidia, germ tube, appressorium and invasive hyphal. The results showed that *MoBZIP3*-GFP could colocalize with NLS-mCherry in the nucleus in these cells (Figure 5A). We also found that the *MoBZIP3* in *M. oryzae* all contains a conserved basic region leucin zipper domain at the C-terminal. However, it also appears non-conserved at the N-terminal of this protein through alignment of amino acid of *MoBZIP3* from Y34, P131 and Guy11 strain (Supplemental Figure S3A). And we further found that the differences in the amino acid at the N-terminal may result in different biochemical activity in the DNA binding. Y34 *MoBZIP3* exhibited higher binding activity to A-box DNA fragment than that from Guy11 (Supplemental Figure S3B,C). The site mutation experiment further showed that *MoBZIP3* specifically binds to A-box DNA fragment (TACGTA) (Supplemental Figure S3D).

Given that lipid catabolism is a major source of glycerol, and glycogen metabolism supplies the energy for turgor pressure during appressorium maturation [24,25], and *MoBZIP3* may function in these processes, we then analyzed expression of several genes which played critical roles in lipid and glycogen metabolism. These included trehalose-6-phosphate (T6P) synthase (TPS1), carnitine acetyl transferase family gene PTH2, isocitrate lyase gene (*ICL1*), peroxin gene (*PEX6*) and multifunctional β-oxidation protein gene (*MFP1*) [23,26,27,28]. We found that expression of these genes was significantly down regulated in Δ*mobzip3* appressorium (Figure 5B).

The results above indicated that *MoBZIP3* may function as a transcription factor in gene expression regulation. And expression of *MoPTH2*, *MoICL1* and *MoPEX6* in Δ*mobzip3* was decreased to 30% at least when compared with WT, indicating that *MoBZIP3* may function with other complex proteins or directly to regulate their expressions. Then electrophoretic mobility shift assays (EMSA) were performed using the biotin-labeled promoters of *MoPTH2*, *MoICL1*, *MoPEX6* and in vitro expressed *MoBZIP3*. The results showed that *MoBZIP3* could directly bind the promoters of these genes (Figure 5C). In addition, the EMSA results further showed that *MoBZIP3* specifically binds to the gene promoters which contains an A-box (Supplemental Figure S3E).

### 2.6. Glycerol Synthesis Is Impaired in Δmobzip3

**Figure 6:**
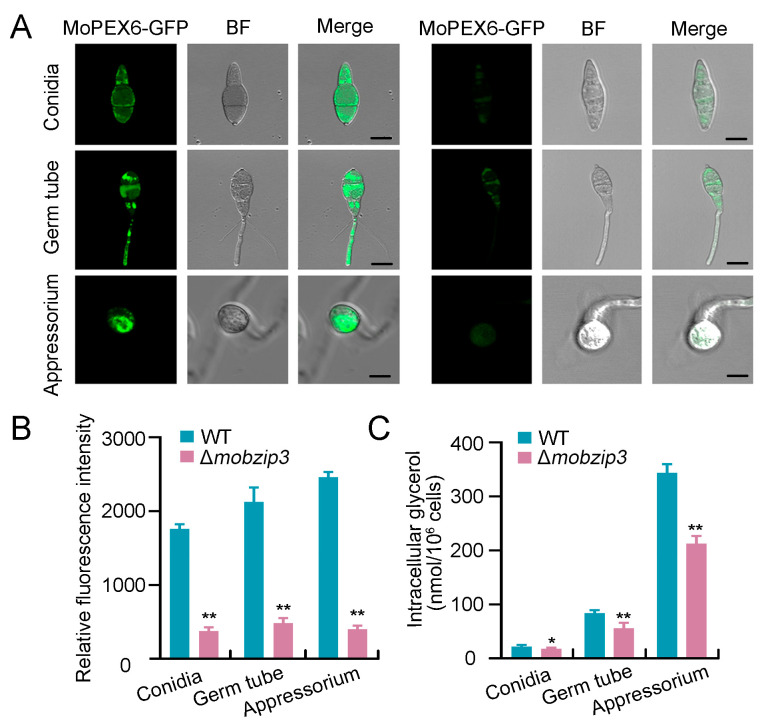
*MoBZIP3* affects glycerol accumulation in appressorium. (**A**), Expression of *MoPEX6* in Δ*mobzip3*. *MoPEX6*-GFP driven by its native promoter was expressed in WT and Δ*mobzip3*. GFP fluorescence were observed under fluorescence microscopy in the *M. oryzae* conidia, germ tube and appressorium. Bar = 10 μm. (**B**), Quantification of GFP fluorescence in (**A**). Error bars represent SD, *n* ≥ 50 cells, ** *p* < 0.01. (**C**), Quantification of glycerol in WT and Δ*mobzip3*. Error bars represent ±SE of three biological replicates and asterisks represent significant differences (* *p* < 0.05 and ** *p* < 0.01).

Glycerol production in appressorium is dependent on complex enzymatic catalysis and cellular events. β-Oxidation is a critical reaction for metabolization of fatty acids in glycerol production. *MoPEX6* was previously reported to function in β-Oxidation of fatty acids [23,29] and in our experiments *MoPEX6* was down regulated in Δ*mobzip3*. To further explore that *MoBZIP3* may function in glycerol metabolism, we validated the expression of *MoPEX6* in Δ*mobzip3*. *MoPEX6*-GFP driven by *MoPEX6* native promoter was expressed in WT and the Δ*mobzip3* strain. Fluorescence observation showed that *MoPEX6*-GFP exhibited as bright spots in WT conidia, germ tube and appressorium. In contrast, the fluorescence intensity in the Δ*mobzip3* strain was much lower (Figure 6A). Quantitative analysis of the fluorescence intensity further supported the conclusion (Figure 6B).

Furthermore, we measured the amount of glycerol in WT and Δ*mobzip3*. The results showed that glycerol is generated rapidly in germ tube and appressorium in WT. However, in Δ*mobzip3*, the amount of glycerol was much less produced when compared with those in the WT (Figure 6C). Together, these results indicated that *MoBZIP3* functions in appressorium glycerol production.

### 2.7. MoSln1 Mediated Appressorium Turgor Formation Is Affected in Δmobzip3

**Figure 7:**
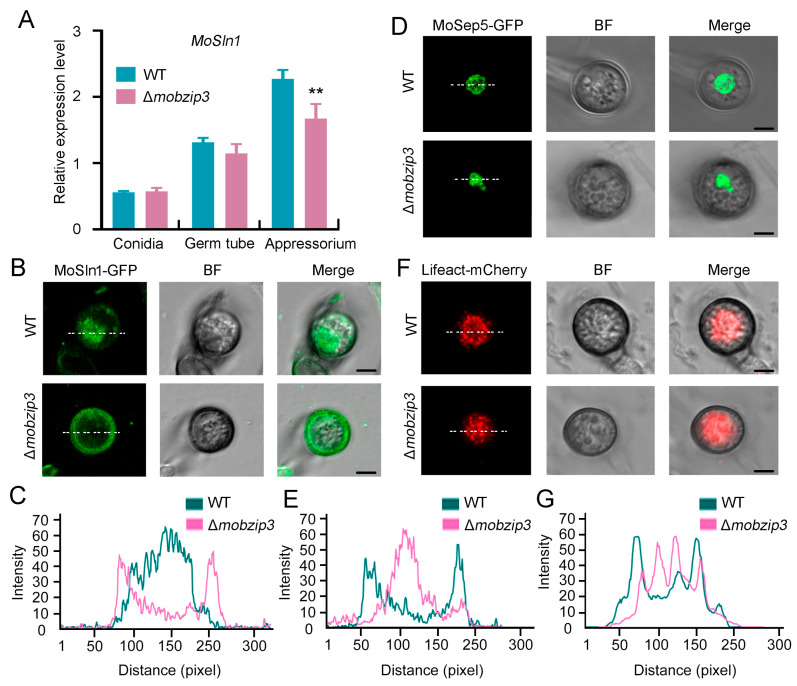
*MoBZIP3* may function in *MoSln1* mediated turgor recognition. (**A**), Quantitative real-time PCR analysis expression of *MoSln1* in WT and Δ*mobzip3*. The experiments were repeated three times with similar results. Error bars represent ±SE of three biological replicates. Asterisks represent significant differences (** *p* < 0.01).Primers used in this experiment were listed in Supplemental Table S1. (**B**,**C**), Distribution analysis of *MoSln1* in *M. oryzae* appressorium. Fluorescence observation of *MoSln1*-GFP (**B**). Line scan analysis of*MoSln1*-GFP (**C**) in (**B**); (**D**,**E**), Distribution analysis of MoSep5 in *M. oryzae* appressorium. Fluorescence observation of MoSep5-GFP (**D**), Line scan analysis of MoSep5-GFP (**E**) in (**D**); (**F**,**G**), Distribution analysis of Lifeact-mCherry labeled actin cytoskeleton in *M. oryzae* appressorium. Fluorescence observation of Lifeact-mCherry (**F**), Line scan analysis of Lifeact-mCherry (**G**) in (**F**). *MoSln1*-GFP and MoSep5-GFP driven by their native promoters were expressed in WT and Δ*mobzip3*. Fluorescence signals were observed at 24 h after conidia germed on glass coverslips under fluorescence microscopy in the WT and Δ*mobzip3* appressorium. At least 50 cells were observed for each fluorescence analysis in this experiment. Bars = 5 μm.

It was revealed that the turgor-sensing histidine–aspartate kinase, *MoSln1*, could enable the appressorium to sense turgor threshold and intrigue the appressorium to penetrate the host cells [9]. And our results indicated that *MoBZIP3* may function in glycerol production, which could generate turgor pressure in appressorium. Thus, we explored whether *MoBZIP3* functions in *MoSln1* initiated turgor-driven plant infection. As *MoBZIP3* is a transcription factor, we first examined the expression level of *MoSln1* in WT and Δ*mobzip3*. The expression of *MoSln1* decreased about 28% in Δ*mobzip3* when compared with WT in the appressorium cell (Figure 7A) indicating that *MoSln1* is also a potential target for *MoBZIP3*.

*MoSln1* could sense the turgor threshold and its subcellular localization is sensitive to changes in turgor. During appressorium maturation, *MoSln1* gradually accumulates to the appressorium pore along with the increasing turgor pressure [9]. We then investigated the distribution of *MoSln1* in Δ*mobzip3*. *MoSln1*-GFP driven by its native promoter was expressed in WT and the Δ*mobzip3* strain. It could be observed that *MoSln1*-GFP accumulated at the appressorium pore at 24 h after conidia germed on glass coverslips. But in the Δ*mobzip3* strain, *MoSln1*-GFP did not accumulate at the appressorium pore. Instead, the *MoSln1*-GFP fluorescence signals were distributed at the periphery of the cell membrane (Figure 7B,C).

Appressorium pore accumulation of *MoSln1* could recruit the actin cytoskeleton assembly as filament actin (F-actin) ring in the infection cell, which is critical for *M. oryzae* penetration into the host cells [30]. We then investigated the actin assembly in Δ*mobzip3*. We expressed the actin scaffold protein, Septin5-GFP and the actin labeling peptide, lifeact-mCherry to label the actin cytoskeleton [30,31]. In the WT appressorium, both the Septin5-GFP and the lifeact-mCherry assemble as ring structures. But in Δ*mobzip3*, abnormal actin structures formed (Figure 7D–G).

In all, these results indicated *MoBZIP3* functions in arppressorium turgor formation and affected *MoSln1*-mediated actin cytoskeleton assembly during *M. oryzae* infection.

## 3. Discussion

*Magnaporthe oryzae* is the causal agent of rice blast, the most destructive disease of cultivated rice worldwide. The devastating fungi depend largely on their adaptability to environments. It has developed its own complex regulatory networks to control developmental processes, pathogenesis and responses to stresses. The BZIP transcription factors are involved in many critical processes in a diverse range of species. In fungi, there are multiple BZIP transcription factors and members of them in *Aspergillus nidulans*, *Aspergillus fumigatus*, *Fusarium graminearum*, *Fusarium oxysporum* which have been shown to be closely linked to various developmental and physiological processes including the fungal metabolism, development, stress responses and virulence [12,32,33,34]. In *M. oryzae*, 22 *BZIP* genes were identified in strain Guy11 and KJ201. And of which, several members were characterized to play crucial functions in development and pathogenesis. For example, *MoBZIP5*, *MoBZIP10*, *MoMeaB* (*MoBZIP12*), *MoHac1* (*MoBZIP18*) and *MoMetR* (*MoBZIP22*) were reported to function associated with many aspects of *M. oryzae* development and host infection [15,16,17]. Considering the diverse critical roles for BZIP proteins in *M. oryzae*, we attempted to identify new functional *BZIP* genes in the Y34 strain, which is isolated from the field and its pathogenicity is different from Guy11 [19]. In this study, we found that in Y34 strain, no BZIP11 and BZIP14 were present in the genome but additional BZIP22 and BZIP23 emerged. we then analyzed expression profiles of the 22 *BZIP* genes in Y34 and found that their expressions were quite different from those in the Guy11 and KJ201, indicating that the *BZIP* genes in Y34 may form a different regulatory network. We further knocked out six *BZIP* genes including *MoBZIP3*, *MoBZIP16*, *MoBZIP17, MoBZIP20*, *MoBZIP23* and *MoBZIP24* which showed high expression levels in *M. oryzae* infection stages (Figure 1). And of which, Δ*mobzip3* exhibited decreased infection capability (Figure 2) indicating that *MoBZIP3* is a positive regulator for *M. oryzae* virulence in *BZIP* family. Virulence of the other five *bzip* mutants obtained in our study did not significantly change though they expressed at a high level in the infection stages. We speculated that some BZIP proteins in *M. oryzae* maybe function in an overlapping manner.

Previous work has shown that knockout of *MoBZIP3* in strain Guy11 and KJ201 did not result in significant changes in the *M. oryzae* development and virulence. In line with their results, in Y34, this gene mutant also exhibits a normal development phenotype (Figure 2A), indicating that *MoBZIP3* may not much function in the fungal development. However, we found that the pathogenesis of Δ*mobzip3* was significantly decreased (Figure 2B–D). We found that the N-terminal of Y34 BZIP3 protein was different from those of Guy11 and KJ201. It lacks 24 amino acids at the N-terminal when compared with the Guy11 (Supplemental Figure S3A,B). *MoBZIP3* from Guy11 possesses a weaker DNA binding activity than the Y34 *MoBZIP3* (Supplemental Figure S3C). Thus, we speculate that no significant changes in Guy11 *MoBZIP3* mutant may associate with its weak DNA binding property. And the amino acid discrepancy in the Y34 strain leads to a stronger DNA binding activity, which may effectively promote the transcription of the targeted genes. Our results suggested that *MoBZIP3* is a new member of functional *BZIP* genes in *M. oryzae* virulence.

The rice blast fungus employs a specialized infection structure called appressorium to penetrate into host plant leaf surface using huge invasive force [35]. The appressorium cell wall which is rich in melanin could act as a rigid structural barrier to help the generation of turgor [6]. The major solute that accumulates in appressorium is glycerol which gradually produces the turgor pressure in the cell [5]. Thus, the generation of glycerol is critical for the appressorium to achieve enough turgor pressure. Prior work showed that lipid degradation is a major route to generate glycerol in appressorium and in this process, the glycogen servers as the energy source [22]. And high levels of triacylcglycerol lipase activity for lipid conversion to both fatty acids and glycerol during turgor generation are proposed in the appressorium. During glycerol generation, fatty acid β-oxidation is required for activation of the glyoxylate cycle and gluconeogenesis, which is significant during plant infection by *M. oryzae* [22,36]. In our study, we found that degradation of lipids and glycogen was significantly decreased in the Δ*mobzip3* appressorium (Figure 4). The *MoTPS1* gene which is involved in glycogen etabolism [26], *MoICL1* gene which functions in glyoxylate cycle catalyzing [28] and *MoPTH2*, *MoICL1*, *MoPEX6* which are critical for fatty acid β-oxidation, were all down regulated in expression in Δ*mobzip3* (Figure 5B and Figure 6A,B). Furthermore, the glycerol accumulation was compromised in the Δ*mobzip3* appressorium (Figure 6C). Considering that *MoBZIP3* localized in the *M. oryzae* nucleus (Figure 5A) and it could directly bind to the promoters of *MoPTH2*, *MoICL1*, *MoPEX6* (Figure 5C), we could conclude that *MoBZIP3* serves as a working transcription factor in *M. oryzae* pathogenesis and its functions were associated with regulation of expression of the glycerol generation related genes.

The appressorium produces turgor of up to 8.0 MPa through accumulating high concentrations of glycerol and other polyols [5]. Accompany with the generation of appressorium turgor, a narrow penetration hypha emerges from the base of the appressorium and breach the cuticle of the rice leaf. In this process, a sensor kinase *MoSln1* could recognize the threshold of turgor and initiate a complex signaling pathway to generate invasive force to cause blast disease [9]. *MoSln1* could change its subcellular localization from the cytoplasm to the appressorium pore in response to turgor increasing, which is critical for its function. In this study, we found that expression of *MoSln1* was down regulated in Δ*mobzip3* (Figure 7A), and *MoSln1* did not change its subcellular localization to the appressorium pore (Figure 7B,C). These indicate that *MoSln1* is a potential target for *MoBZIP3*. Prior work showed that *MoSln1* could recruit septin proteins to the appressorium pore to orchestrate a toroidal network of F-actin, which governs the penetration of the appressorium [30]. But in our study, we found that both the Septin5-GFP and the F-actin ring were all deformed in Δ*mobzip3* (Figure 7D–G). Thus, we concluded that *MoBZIP3* could directly regulate the expression of glycerol synthesis-related genes, and meanwhile, it may also have a function in *MoSln1*-mediated turgor sensing and penetration.

## 4. Materials and Methods

### 4.1. Plant and Fungal Strain Growth Conditions

All *M. oryzae* strains used in this study were derived from the wild-type strain Y34 (kindly provided by Prof. LiHuang Zhu, Institute of Genetics and Developmental Biology, Chinese Academy of Sciences). All strains were cultured on liquid or solid completed medium for growth and straw rice bran medium for conidia generation at 28 °C in the dark. Mycelia were harvested from liquid CM and used for genomic DNA and RNA extractions. Y34 susceptible rice (*Oryza sativa* cv. Nipponbare) were grown at 28 °C under 16 h light and 8 h dark condition and used for fungal infection analyses.

### 4.2. Targeted Gene Deletion and Plasmid Construction

The *BZIP* gene deletion mutants were generated using the standard one-step gene replacement strategy [16,31]. To construct plasmids expressing MoBZIP3-GFP, MoPEX6-GFP, MoSep5-GFP, ~1.5-kb native promoter region from the *M. oryzae* genome were amplified and cloned into the pKNTG binary vector [37]. All constructs were cloned by homologous recombination (ClonExpress MultiS One Step Cloning Kit, Vazyme Biotech, Nanjing, China, C112); all primers with restriction enzyme sites are listed in the Supplemental Table S1.

### 4.3. RNA Extraction and qRT-PCR Analysis

Total RNA was extracted from the samples using a Total RNA Purification kit (TransGen, Fuzhou, China, ET101-01) according to the manufacturer’s protocol. For the gene expression analysis in leaf blades, total RNA was isolated from three 5-cm long leaf sections per plant spotted with conidia suspension. Data were normalized to the expression levels of Actin in *M. oryzae* [20]. qRT-PCR was performed using Perfect Start Green qPCR SuperMix (TransGen, Fuzhou, China, AQ601) with a Bio-Rad CFX96 real-time PCR detection system. Primer sequences are listed in Supplemental Table S1.

### 4.4. Pathogenicity Analysis Assay

The wild-type *M. oryzae* strains and each mutant were cultured on rice bran medium for 7 days. For spray inoculation of conidia, prepare conidia suspension 1 × 10^5^ spores/mL in a 0.02% (*w*/*v*) tween-20 solution and spray it evenly on the 2–3 weeks rice leaves with sprayer. The inoculated plants were grown in a growth chamber at 28 °C, with high humidity in the dark for the first 24 h, followed by a 12-h/12-h light (20,000 Lux)/dark cycle. The development disease lesions were observed at 4–5 days after inoculation [38].

For plant sheath cells penetration assays, the conidia suspension of GFP labeled WT, Δ*mobzip3* and the complemented strain was injected into the rice leaf sheath under high humidity, and the development and infection status of the rice blast fungus in the leaf sheath cells were observed under microscopy (Zeiss LSM880, Oberkochen, Germany, with a 40× objective) [20].

### 4.5. Protein Expression and Purification in Escherichia coli 

ORF sequence of *MoBZIP3* was cloned into the Pet-28a vector between the BamH1 and HindIII sites to generate. The fusion constructs were introduced into BL21 (DE3). Bacteria containing the plasmid was grown in Luria–Bertani (LB) medium containing 100 mg/mL Kan at 37 °C to OD600 = 0.6. Expression of the fusion protein His-MoBZIP3 was induced by the addition of 0.5 mM isopropyl b-D-1 thiogalactopyranoside (IPTG) and incubation at 16 °C for 14 h. Then His-tagged MoBZIP3 proteins were purified using nickel-nitrilotriacetic acid resin following procedures described by the manufacturer (TransGen, DP101).

### 4.6. Electrophoretic Mobility Shift Assay

Light Shift Chemiluminescent EMSA Kit (Beyotime Biotechnology, Shanghai, China, No. 3308) was used in this experiment. Biotin was labeled at 3′ end and 5′ end of primers for each gene. The biotin-labeled DNA from upstream of the ORF for each gene were obtained through PCR. The detailed procedure of EMSA follows the manufacturer’s instructions. Photos obtained by ChemiDocTM Imaging Systems (Bio-Rad Laboratories, Hercules, CA, USA, ChemiDoc MP).

### 4.7. Lipid Droplets and Glycogen Staining

Lipid droplets and glycogen staining mainly followed the method described previously [39]. The suspension of conidia (20 μL) was cultured on a hydrophobic membrane to induce formation of appressorium at 25 °C in the dark for 0 h, 2 h, 8 h, 16 h, and 24 h, respectively. Nile red staining was carried out by using the staining solution (final concentration 1 mM) for 30 min. Then the distribution of lipid droplets was observed under fluorescence microscopy (Zeiss LSM880, with a 40×objective). Similarly, glycogen was stained with I2/KI solution (1 mM) for 1 min. And the images were obtained with Zeiss microscopy (Zeiss A1). To analyze the glycogen, image processing and measurements were performed using ZEN software (Zeiss 2.3 lite). Images were selected using the ‘rectangle’ tool and the mean value of the selected region was calculated using the “Measure” plugin. The experiment was repeated 3 times, and 100 conidia were counted for each treatment.

### 4.8. Accession Numbers

Sequence data for the genes described in this study can be found in the GenBank/EMBL data under the accession numbers: MoBZIP1 (ELQ37252.1), MoBZIP2 (ELQ38751), MoBZIP3 (ELQ37958.1), MoBZIP4 (ELQ32319.1), MoBZIP5 (ELQ36319), MoBZIP6 (ELQ42426.1), MoBZIP7 (ELQ34568.1), MoBZIP8 (ELQ39141.1), MoBZIP9 (ELQ41744.1), MoBZIP10 (ELQ42748.1), MoBZIP12 (ELQ44954.1), MoBZIP13 (ELQ40875.1), MoBZIP15 (ELQ34378.1), MoBZIP16 (ELQ35717.1), MoBZIP17 (ELQ37205.1), MoBZIP18 (ELQ33334.1), MoBZIP19 (ELQ33489.1), MoBZIP20 (ELQ41552.1), MoBZIP21 (ELQ44526.1), MoBZIP22 (ELQ36618.1), MoBZIP23 (ELQ40983), MoBZIP24 (ELQ38771.1), MoSep5 (ELQ40999.1), MoSln1 (ELQ34371.1), MoPEX6 (ELQ38871.1).

## Data Availability

The data presented in this study are available in “*Magnaporthe oryzae* Transcription Factor *MoBZIP3* Regulates Appressorium Turgor Pressure Formation during Pathogenesis”.

## Supplementary Materials

The following supporting information can be downloaded at: https://www.mdpi.com/article/10.3390/ijms23020881/s1.
