# Supplementary material for: Magnaporthe oryzae Transcription Factor MoBZIP3 Regulates Appressorium Turgor Pressure Formation during Pathogenesis
Source: Int J Mol Sci. 2022 Jan 14;23(2):881. doi: 10.3390/ijms23020881 (PMC8778449; doi:10.3390/ijms23020881)
Supplement: Supplementary file 1 [file ijms-23-00881-s001.zip › ijms-1526049-supplementary.pdf]

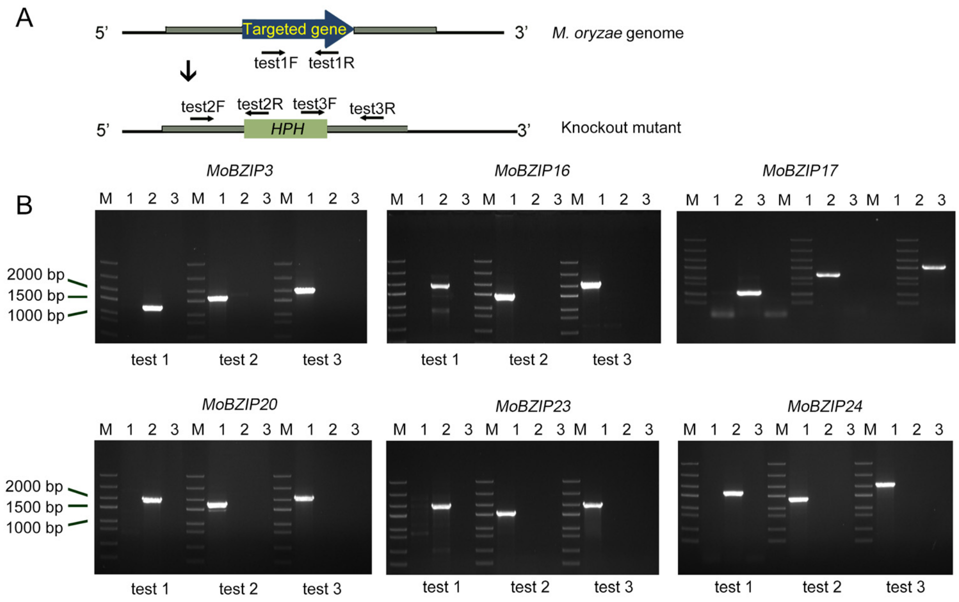

Figure S1. *Targeted gene deletion in M. oryzae*

A, Schematic illustration of the gene deletion of *bZIP* genes in *M. oryzae*. (b) PCR analysis of the *bZIP* gene deletion mutants with the indicated four primer pairs. Lanes 1, 2, and 3 indicate the WT, the mutant and the mock control.

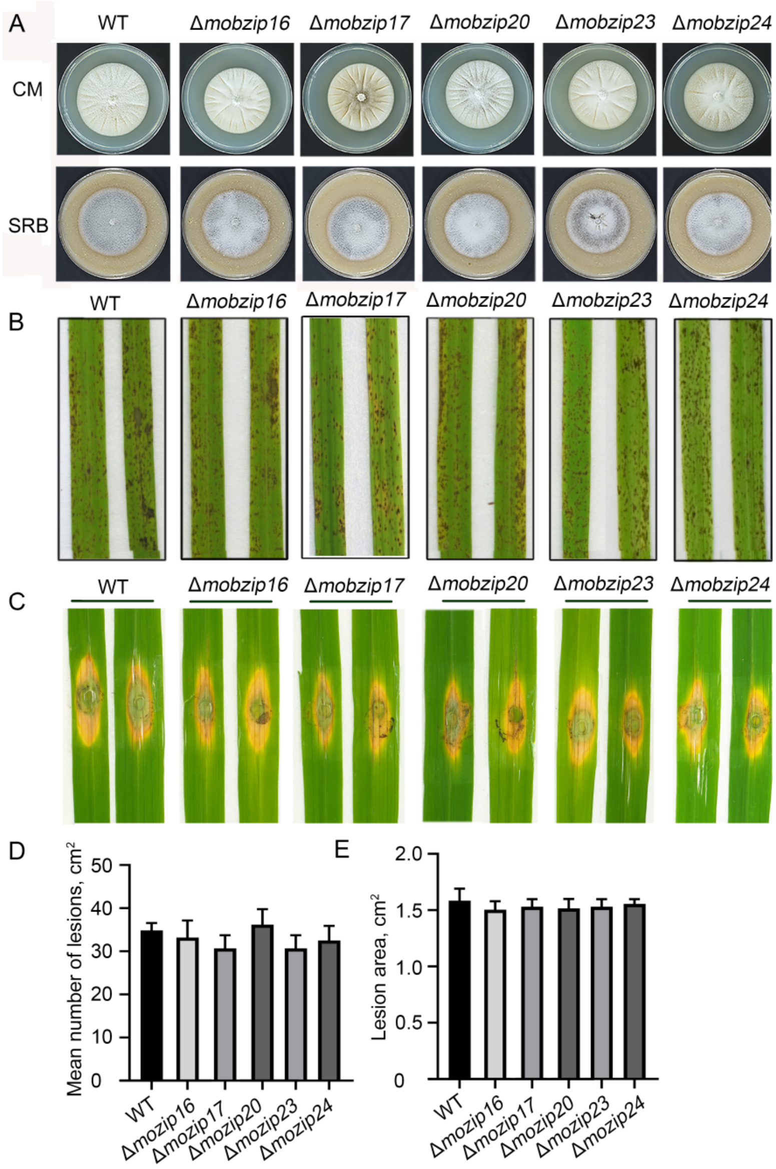

Figure S2. *Pathogenesis analysis of mutants of bZIP genes*

A, Same numbers of conidia of WT and bZIP gene mutants (Δ*mozip16*, Δ*mozip17*, Δ*mozip20*, Δ*mozip23* and Δ*mozip24*) were cultured on the completed mediate (CM) and straw rice bran (SRB) medium. Images showing seven-day-old cultures of the indicated *M. oryzae* on the two types of mediums. B and C, Pathogenesis analysis of these indicated *M. oryzae* using conidia spraying (B) or punched (C) inoculated methods to infect rice leaves (*O. sativa cv.* Nipponbare). Images were obtained from 5 days after inoculation. The experiments were repeated three times with similar results. D and E, Quantification of the lesion numbers (D) and area (E) of the rice leaves

shown in (B) and (C), respectively. Error bars represent SD (n = 20).

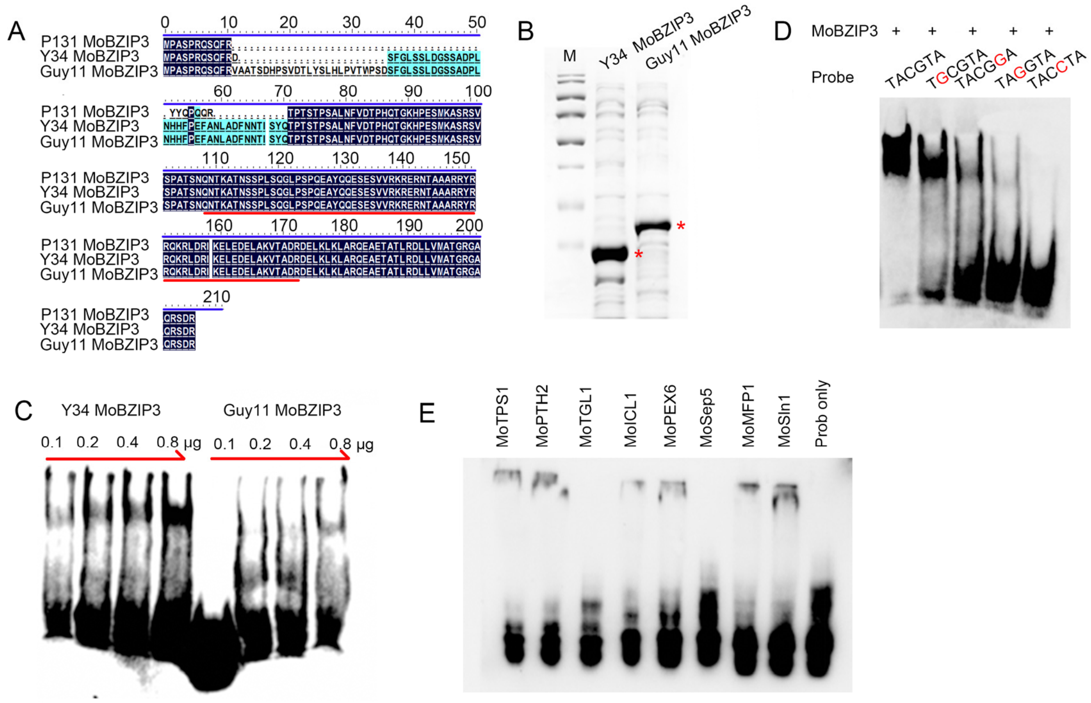

Figure S3. *Biochemical analysis of MoBZIP3*

A, Amino acids of MoBZIP3 of *M. oryzae* strain P131, Y34 and Guy11. The amino acids highlighted with a red line indicate the conserved basic region leucin zipper domain; B, *In vitro* expressed MoBZIP3 from Y34 and Guy11; C, EMSA showing the DNA binding activity of MoBZIP3 from Y34 and Guy11. Increasing amount of Y34 and Guy11 MoBZIP3 proteins were incubated with Bio-A-box; D, Y34 MoBZIP3 binds the A-box or the mutants of A-box DNA fragments, the red bases indicate the site mutations of the DNA fragments; E, Y34 MoBZIP3 binding promoters of *MoTPS1*, *MoPTH2*, *MoTGL1*, *MoICL1*, *MoPEX6*, *MoSep5*, *MoMFP1* and *MoSln1*. Promoters of *MoTPS1*, *MoPTH2*, *MoICL1*, *MoPEX6*, *MoMFP1* and *MoSln1*contain an A-box.

Table S1. Primers used in this study.

The nucleotides highlighted in red indicate the enzyme site for construction.

MoBZIP3-ORF-F	ATGCCGGCCTCACCAAGACAG
MoBZIP3-ORF-R	CTACCTATCAGAACGTTGCGC
pKNTG-MoBZIP3- GFP-F	CGACGGCCAGTGCCAAGCTTACTTACATATCGCCGCCTC
pKNTG-MoBZIP3-GFP-R	GCCCTTGCTCACCATCCCGGGCCTATCAGAACGTTGCGC
MoBZIP3-KO-HPT-5'F	GGTGGCGGCCGCTCTAGAACTTACATATCGCCGCCTC
MoBZIP3-KO-HPT-5'R	CAAAAATGCTCCTTCAATCTAGAGGCACTCAGACAGGGTA
MoBZIP3-KO-HPT-3'F	GGGTTCGCAAAGATAAAAGCTTAGGTGGGATCCGATAGAT
MoBZIP3-KO-HPT-3'R	GGTCGACGGTATCGATAAGCTTCGATGATATTTGCACAACG
MoBZIP3-PET-28a-F	GACAGCAAATGGGTCGCGGATCCATGCCGGCCTCACCAA G
MoBZIP3-PET-28a-R	GCTCGAGTGCGGCCGCAAGCTTCTACCTATCAGAACGTTG
Δ *mobzip3*-test-1	CGTTAAGCGA TATGTCGATG C
Δ *mobzip3*-test-2	CATCTCAACGCTCTATTCGC
Δ *mobzip3*-test-3	ATGCTCGCAAGGAGGGGTC
Δ *mobzip3*-test-4	TCAGATGTGGCCATAGCTCG
MoBZIP16-KO-HPT-5'F	GGTGGCGGCCGCTCTAGACGTAAGGCAT CTGTTATGCT C
MoBZIP16-KO-HPT-5'R	CAAAAATGCTCCTTCAATCTAGATGATGCGACTGGCGATA G
MoBZIP16-KO-HPT-3'F	GGGTTCGCAAAGATAAAAGCTTTGTCGACCTTTTTGGTCC C
MoBZIP16-KO-HPT-3'R	GGTCGACGGTATCGATAAGCTTAAGGACACGGAAGCTAAC
Δ *mobzip16*-test-1	CCATCTCATC CAGAGCAACG
Δ *mobzip16*-test-2	GAGAGATCCATTGACTCAGG
Δ *mobzip16*-test-3	ATGAGCAGGAGCACCATCAA
Δ *mobzip16*-test-4	CTAAGACATGAAATTCAAAG
ELQ40875’	ATGAGGACCTC ATCTCGTAGC
ELQ40875’	CATCTAAGATTCAGCAACCC
ELQ40875-F	ATGACGGGCTCCCGAACAAG
ELQ40875-R	TCAATTGCCAGATCCAAACC
MoBZIP20-KO-HPT-5'F	GGTGGCGGCCGCTCTAGAACATTTTCAA TCCGGGCCATG
MoBZIP20-KO-HPT-5'R	CAAAAATGCTCCTTCAATCTAGACGGTGGCTCGACAATCG A
MoBZIP20-KO-HPT-3’F	GGGTTCGCAAAGATAAAAGCTTAGTCTGAGTCTGTCTGGG
MoBZIP20-KO-HPT-3'R	GGTCGACGGTATCGATAAGCTTTGGTATGTAGCAATTGCAC
Δ *mobzip20*-test-1	TTCTAGTGCA AACCGGACAG
Δ *mobzip20*-test-2	CAAGGTATTGCCCGAACT

Δ *mobzip20*-test-3	ATGGCTTCCACATTTCAAAT
Δ *mobzip20*-test-4	TCATTCATGTGCGTCGTTGT
MoBZIP23-KO-HPT-5'F	GGTGGCGGCCGCTCTAGAGGCCAAGGTA CAGTTCAGTAG
MoBZIP23-KO-HPT-5'R	CAAAAATGCTCCTTCAATCTAGATGTGAATAGACTTCTAGA
MoBZIP23-KO-HPT-3’F	GGGTTCGCAAAGATAAAAGCTTACCGAACGATTTATACTC G
MoBZIP23-KO-HPT-3'R	GGTCGACGGTATCGATAAGCTTCGTATCGGAGTATTTTGGA T
Δ *mobzip23*-test-1	CCAACTCTGAAAATAAGCACG
Δ *mobzip23*-test-2	CGAAGTTCCC TGTGTGATAT G
Δ *mobzip23*-test-3	ATGTCCATCGACAGAAAAGA
Δ *mobzip23*-test-4	TCACCTCATGTTGCTTGTGTC
MoBZIP24-KO-HPT-5'F	GGTGGCGGCCGCTCTAGATACGACGTGGCGTAGCTTCT
MoBZIP24-KO-HPT-5'R	CAAAAATGCTCCTTCAATCTAGACTTGTCTGCCATACCTGT
MoBZIP24-KO-HPT-3’F	GGGTTCGCAAAGATAAAAGCTTTACCTCGGCCACTTCAAA
MoBZIP24-KO-HPT-3'R	GGTCGACGGTATCGATAAGCTTGGAGCATTGACTTCAATCT
pKNTG-PTS1-GFP-F	CGACGGCCAGTGCCAAGCTTATATGCCTTAACCG ACCTC
pKNTG-PTS1-GFP-R	GCCCTTGCTCACCATCCCGGGCAGCTTCGACATTGTGATG A
pKNTG-MoSLN1-GFP-F	CGACGGCCAGTGCCAAGCTTT GAATGTTGTT CCGCCACCT
pKNTG-MoSLN1-GFP-R	GCCCTTGCTCACCATCCCGGGCGTCGCCACAGCAGCACCG
pKNTG-MoSEP5-GFP-F	CGACGGCCAGTGCCAAGCTTGTTGGAAAGCCGAAATGTC
pKNTG-MoSEP5-GFP-R	GCCCTTGCTCACCATCCCGGG GTTGCCGTCT TCCCCGTTT

Primers used for quantitative RT-PCR

MoActin-F	AG TCCAGGTTAT CACTCACA
MoActin-R	GATTCCAGGTCCATATTCTTGG
RT- MoBZIP3-F	CGACAAACTCATCTCCCTT
RT- MoBZIP3-R	GACCAACAAATCCCTCAG
ELQ41552.1-F	ACCAGTCCCTGCCGTTCT
ELQ41552.1-R	GAGCTCGACCAGCTGCGCAG
ELQ40875.1-F	GCTGACACATACAAGACGCT
ELQ40875.1-R	CAAACCTGACATCAAACCC
ELQ34378.1-F	GCAACGACCTTCTCAGTCA
ELQ34378.1-R	GCATACAGCCCAATGTCA
ELQ33489.1-F	ATCGCATCTCGGAACTTG
ELQ33489.1-R	AACAACTTCTCACAGCAGCA
ELQ44526.1-F	AAGAAGGCTAAATGCTCTGG
ELQ44526.1-R	CTTGATGAACCAAATCCCAC
ELQ34568.1-F	GCTCGTTGATGCTGCTGAT
ELQ34568.1-R	TGTCTTTCGTGGTGCGTCT
ELQ42748.1-F	ATTCCTCGGGATGTGGTCA

ELQ42748.1-R	GCATAACAAGCCAGATTCAACC
ELQ42426.1-F	CTGTGTCCTCAGCCTCTACA
ELQ42426.1-R	TTTATGGGTCCGACTTCG
ELQ37252.1-F	TGATGGAGGAGTTCGAGGCT
ELQ37252.1-R	CAGGGACCTCATGCTTGGA
ELQ32319.1-F	ACCTATGAGAAGCCCACGA
ELQ32319.1-R	AGCCAGTAGCAAACCGAGA
ELQ36319.1-F	GCCGAAATCAGCAACAGT
ELQ36319.1-R	AAAGGGGAGTAGAGGCATAG
ELQ39141.1-F	TCAATATCCGAGAGCGCATG
ELQ39141.1-R	GACAAAATTTGCCGTCCGTC
ELQ44954.1-F	GCTACACATACCACCAGTGC
ELQ44954.1-R	TGAGAACCGTAATCGGGT
ELQ43059.1-F	CACCAT CTGCCTGGGC AGC
ELQ43059.1-R	GAGATGGTAC ACTGTTCAAG
ELQ38751.1-F	TGAGCGTGACCACTTCAA
ELQ38751.1-R	CGTGGATGTTCTCGTGTTT
ELQ35717.1-F	CACGACAGCAACATACCAA
ELQ35717.1-R	GCCATAAACGCCTTGTGT
ELQ37205.1-F	AAAGACATTACTCCTTGCCCAC
ELQ37205.1-R	TTCGTTCTATGAATACCGCCTC
ELQ3334.1-F	TGACGCCAAGAAGACCTCT
ELQ3334.1-R	GAATGGGTTTGACCAGCA
ELQ40983.1-F	AGCGACCCACAGTATCTGAG
ELQ40983.1-R	ATGCCTCCAGTTCTTCGGT
ELQ38771.1-F	TGGGCAACTATGGCTACA
ELQ38771.1-R	GATAAAGGAGTGGTCGGGT
ELQ41744.1-F	CACGATTGAGCACCTTGA
ELQ41744.1-R	AGAGTGGGTATTCATCAGTCG
RT-MoPEX6-F	GAGGAGGAAGATAGGCAAGA
RT-MoPEX6-R	TGAGATTTACCCGTCAGAGTC
RT-MoPTH2-F	TTCAACATCGTCTCCAAGG
RT-MoPTH2-R	AAGTCAAAGGAAACCCAGC
RT-MoMFP1-F	TCAAGAACATCAAGGTCCG
RT-MoMFP1-R	TCGGTATTTCAGCCTACAGA
RT-MoICL1-F	GATGTCGTCAAGCACCAA
RT-MoICL1-R	TGTCTATTGCGGAGTCAGC
RT-MoTPS1-F	TTCGTCGCCGAGCTCAATAG
RT-MoTPS1-R	GGCACGCAAATAGGCAGATG
RT-MoTGL1-F	CATTCAACGCCATACCAGCG
RT-MoTGL1-R	GAGGCAAACGGACCAGAGAA
RT-MoSLN1-F	ACGGGACCAAGTCATCAGT
RT-MoSLN1-R	CCAGACGACAGACGAGATAA

Primers used for EMSA

A-box-EMSA-F-Biotin	ATTACGTATGTACGTATC
A-box-EMSA-R-Biotin	GATACGTACATACGTAAT
A-box-EMSA-F	ATTACGTATGTACGTATC
A-box-EMSA-R	GATACGTACATACGTAAT
MoTPS1-Biotin-F	TTAGCTGGAATAAGGCGGGCA
MoTPS1-Biotin-R	TTGTACCAAGAGATGCAAAGC
MoPTH2-Biotin-F	GAAATCTCCTATGCTCACAAG
MoPTH2-Biotin-R	CACAAAAAAACAACCTTCCA
MoTGL1-Biotin-F	CGCTTATTACCCCTTTGTCG
MoTGL1-Biotin-R	CATTAAGTGC AGGGCTAGAA G
